# Parasite Screening in Wild Passerines: Enhancing Diagnostic Approaches in Wildlife Rehabilitation Centers

**DOI:** 10.3390/ani14243664

**Published:** 2024-12-19

**Authors:** Catarina Ferreira Rebelo, Alicia Carrero Ruiz, Alberto Alvarado-Piqueras, Fernando González González, Luís Madeira de Carvalho

**Affiliations:** 1 Centre for Interdisciplinary Research in Animal Health (CIISA), Faculty of Veterinary Medicine, University of Lisbon, Av. da Universidade Técnica, 1300-477 Lisbon, Portugal; 2 Associate Laboratory for Animal and Veterinary Sciences (AL4AnimalS), Av. da Universidade Técnica, 1300-477 Lisbon, Portugal; 3 Group for the Rehabilitation of Native Fauna and Its Habitat (GREFA), C. Monte del Pilar, s/n, 28220 Majadahonda, Madrid, Spain; aliciacarrero.ruiz@gmail.com (A.C.R.); alberto@grefa.org (A.A.-P.); fgonzalez@grefa.org (F.G.G.); 4 Departmental Section of Pharmacology and Toxicology, Faculty of Veterinary Science, University Complutense of Madrid, Av. Puerta de Hierro, s/n, Moncloa—Aravaca, 28040 Madrid, Spain

**Keywords:** birds, coccidia, helminths, parasites, Passeriformes, passerines, wild, Spain

## Abstract

The order Passeriformes is the richest and most abundant group of birds, but despite numerous parasites being identified in wild birds, this order has received limited focus. This study analyzed 17 passerines admitted to the Grupo de Rehabilitación de la Fauna Autóctona y su Hábitat (GREFA), a wildlife rehabilitation center in Spain, during October to December 2022. Necropsies were conducted to determine the presence of parasites, and intestinal contents were analyzed using fecal smear, flotation and sedimentation techniques and the McMaster method. Sixteen passerines (94.1%) were positive for parasites. Identified species included *Monojoubertia microhylla* and the genera *Ornithonyssus* sp., *Diplotriaena* spp., *Serratospiculum* sp., *Porrocaecum* sp., *Capillaria* spp., *Syngamus* sp., *Strongyloides* sp. and *Brachylecithum* sp., besides cestodes and coccidia. The comparative analysis of parasitological diagnostic techniques showed that the Willis flotation technique was effective for detecting coccidia. However, to obtain more accurate results for other parasites, it is important to complement this technique with direct examination or sedimentation techniques. Among the 12 passerines positive for coccidia, oocyst counts per gram of intestinal contents ranged from 100 to 30,450, with a median of 7350. This study provides valuable insights into the parasitic fauna of Passeriformes, serving as a cornerstone for future research and enhancing our understanding of these avian species.

## Simple Summary

The birds of the order Passeriformes have seen limited advances in veterinary medicine, and knowledge about their parasitic fauna remains limited. Wildlife rehabilitation centers often receive injured or debilitated passerines, providing essential care for their recovery. However, many of these centers do not prioritize parasite screenings or necropsies for passerines, despite the valuable insights these procedures can provide. This study addresses this gap by offering crucial information on the parasites of wild passerines admitted to a wildlife rehabilitation center and introducing simple, cost-effective diagnostic methods that can be applied in this context, also serving as a foundation for future research. Our findings revealed that 94.1% (16 out of 17) of the passerines sampled were positive for parasites, including genera identified for the first time in Spain, demonstrating the importance of implementing and refining parasitological diagnostic techniques.

## 1. Introduction

The order Passeriformes is the richest and most abundant group of birds, comprising approximately 6500 species, with two out of every three bird species belonging to this order [1]. Wildlife rehabilitation centers often receive injured or debilitated passerines, providing essential care for their recovery. However, the combination of captivity and the birds’ weakened state can exacerbate infections or promote their spread, particularly for parasitic ones. These infections pose a significant threat to their health and may compromise their successful release back into the wild. In this context, parasitological evaluation is crucial, both to prevent and manage infections in wild birds kept in captivity and to enhance the success of conservation efforts.

Parasitism inevitably depletes resources from the host. Wild birds are affected by a variety of parasites that can cause clinical signs or lesions, increase susceptibility to predators or even lead to death [2]. Additionally, birds act as significant reservoirs of parasitic agents that can affect a wide range of taxa, including humans and other mammals [3]. Migratory species, such as *Turdus* spp., can easily transport parasites across different regions, potentially spreading infections over vast distances [4].

In rehabilitation contexts, birds are often more vulnerable to parasitic infections due to a weakened immune system, resulting from stress, injury, disease or malnutrition [2]. While these infections are typically asymptomatic in healthy hosts, they can cause severe and harmful effects in debilitated birds, those with concurrent diseases and younger individuals [5]. Moreover, parasites can have significant population-level impacts, such as increasing chick mortality and reducing adult reproductive success [6].

Despite the identification of numerous parasites in wild birds [2], knowledge about parasitic infections in passerines remains largely fragmented, with far fewer documented cases than in other avian orders. This knowledge gap exists partly because studies on wild bird parasitology tend to focus on raptors or larger birds, leaving a significant void in our understanding of parasite fauna in passerines. Additionally, wildlife rehabilitation centers often do not prioritize parasitological assessments or necropsies in passerines, a situation further complicated by the challenges of sample collection.

Diagnosis of parasitic infections in birds can be achieved through coprological methods to detect eggs in feces, through blood samples or through endoscopy. However, the most accurate diagnosis is often obtained *post mortem* through necropsy [7]. Notably, to date, coprological analyses in passerines have primarily employed the Willis flotation technique [8] and have not explored intestinal contents as a matrix for research.

This study aims to address these gaps by employing practical, low-resource yet effective diagnostic methods in passerines, including necropsies, to develop and adapt these methods specifically for passerine species. By gaining a more comprehensive understanding of parasitic infections in this diverse order, the study seeks to contribute to better management and treatment strategies in rehabilitation centers, ultimately enhancing conservation efforts.

## 2. Materials and Methods

### 2.1. Sample and Data Collection

Between October and December 2022, necropsies were conducted on 17 wild passerines admitted to the Grupo de Rehabilitación de la Fauna Autóctona y su Hábitat (GREFA) in Majadahonda, Madrid, Spain. The primary objective was to detect parasites and collect intestinal contents for coprological analysis. The sample was heterogeneous, encompassing individuals from 12 different species with varied causes of admission and treatments administered.

Corpses were collected either after death during hospitalization or following euthanasia in cases where severe injuries precluded recovery.

After confirmation of death, cadavers were placed in labeled bags and transported to a refrigeration unit maintained at 4 °C. The cadavers were refrigerated for no more than 48 h until the post mortem examination was conducted. Necropsies were performed using magnifying glasses and the smallest available surgical instruments at GREFA.

The parasitological study was conducted only after the death of the passerines. External and internal macroscopic parasites were preserved in 70% ethanol for subsequent identification. Intestinal contents were collected at the end of the necropsy to prevent contamination. Due to the limited amount of material, all available intestinal contents (from both the small and large intestines) were collected for coprological analysis. Samples were kept refrigerated at 4 °C in labeled collection tubes and analyzed on the day of necropsy or the following day.

### 2.2. Coprological Techniques

Coprological techniques were selected based on the limited intestinal contents available from these bird species and the need for quick, simple and cost-effective diagnostic methods that require minimal equipment. Initially, a direct fecal smear [8] was performed on all samples, followed by concentration techniques such as Willis flotation [8,9] and sedimentation by centrifugation [9,10] and then the quantitative McMaster method [9] for parasitic forms.

The direct fecal smear was used to detect nematode, trematode and cestode eggs; various protozoan stages (such as oocysts and trophozoites) and larvae [8,10], e.g., *Strongyloides* sp. To prepare the smear, a few drops of saline and a proportional amount of intestinal contents were placed on a slide, gently homogenized with a loop and covered with a coverslip. Excess saline was removed, and the smear was examined under an optical microscope with magnifications of 40×, 100× and 400×.

For the Willis flotation technique, a saturated solution of magnesium sulfate was used to float parasitic forms that were less dense than the solution, such as oocysts of protozoa and eggs of nematodes and cestodes [8]. To prepare the sample, 2 grams of intestinal contents, complemented with cloacal content if necessary, were mixed with 28 mL of magnesium sulfate solution. After filtration, slightly more than 5 mL of the suspension was transferred into a 5 mL test tube, allowing a meniscus to form. A coverslip was placed over the top of the tube, and after 15–20 min, it was transferred to a slide for examination under an optical microscope with magnifications of 40×, 100× and 400×.

The sedimentation technique (with centrifugation) was used to detect eggs of trematodes, acanthocephalans and some nematodes and cestodes that are too dense to float [8,11]. An additional 4 mL of the previous suspension of intestinal contents and magnesium sulfate was transferred into a 5 mL test tube (ensuring that it was not filled to the top) and centrifuged at 447.5× *g* for 2–3 min using a Nahita model 2615 centrifuge. The supernatant was discarded, and a drop of the sediment was mixed with methylene blue dye on a slide for microscopic examination with magnifications of 40×, 100× and 400×.

The McMaster method, based on flotation principles, was used to quantify eggs or oocysts per gram of feces, enabling the detection of parasitism by coccidia, certain nematodes and, less commonly, some cestodes [11,12]. The remaining portion of the previously described solution prepared with 2 g of intestinal content and 28 mL of saturated magnesium sulfate solution (as part of it was previously used for the Willis flotation and sedimentation techniques) was filtered into a graduated beaker and then loaded into the McMaster chambers. After a 5 min settling period, the number of eggs or oocysts was counted under a Motic BA210E microscope at 100× magnification. The results were expressed as eggs per gram (EPG) or oocysts per gram (OPG) of intestinal contents.

If McMaster results were negative but qualitative methods were positive, counts were considered below 50 EPG for that specific parasite, which is the detection limit of this method [9].

### 2.3. Parasite Identification

The identification of external parasites and macroscopic internal parasites detected during necropsy was carried out using a wide range of bibliographic references and specific articles [2,13,14,15,16,17,18,19,20,21,22,23]. Through observation under a microscope and magnifying glass, it was possible to recognize distinctive morphological characteristics, which, together with their location in the passerine, enabled the identification of adult parasites to the genus level.

The identification of eggs and oocysts detected through coprological techniques was based on their morphology, considering the host, the geographical location and the presence of adult parasites during necropsy. These results were obtained through consultation of an extensive bibliography [2,8,22,24,25,26,27,28,29,30,31].

### 2.4. Statistical Analysis

Descriptive statistics were calculated using Microsoft Office Excel 2019.

## 3. Results

### 3.1. Sample

For this study, 17 passerines admitted to a wildlife rehabilitation center in Spain, GREFA, were analyzed over the months of October, November and December 2022. The Passeriformes included in the sampling comprise a total of 12 distinct species, with most of these species (9/12; 75%), namely, the long-tailed tit (*Aegithalos caudatus*), meadow pipit (*Anthus pratensis*), European greenfinch (*Carduelis chloris*), short-toed tree-creeper (*Certhia brachydactyla*), European robin (*Erithacus rubecula*), common chaffinch (*Fringilla coelebs*), coal tit (*Parus ater*), house sparrow (*Passer domesticus*) and spotless starling (*Sturnus unicolor*), represented by only one individual each. Additionally, there were two Eurasian blackcaps (*Sylvia atricapilla*), three Eurasian blackbirds (*Turdus merula*) and three song thrushes (*Turdus philomelos*), corresponding to 12% (2/17), 18% (3/17) and 18% (3/17) of the total sample, respectively.

**Figure 1:**
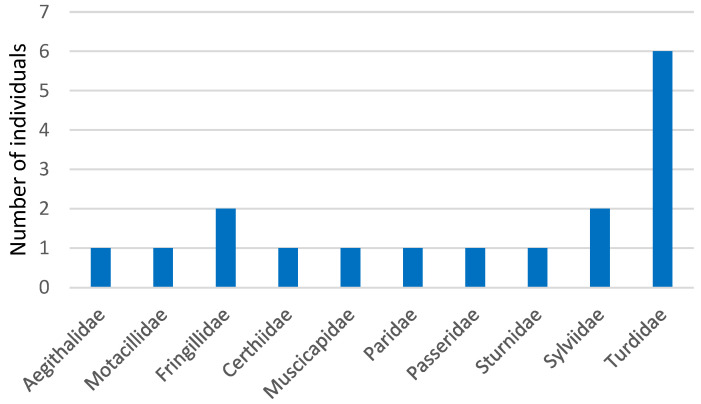
Distribution of passerine sample by family.

As shown in Figure 1, the most represented families of passerines were Fringillidae, Sylviidae and Turdidae.

Seventy-six percent (13/17) of the passerines died during handling or while hospitalized, whereas 24% (4/17) of the sample underwent euthanasia. It was determined that 47% (8/17) of the passerines died within one day of hospitalization, 29% (5/17) within less than a day, 12% (2/17) after two days, 6% (1/17) after five days and, finally, 6% (1/17) after six days.

### 3.2. Qualitative Parasitological Results

Among the 17 passerines analyzed, 16 (94.1%) were found to be positive for the presence of parasitic forms in at least one of the techniques used, including parasites detected during necropsy. The house sparrow was the only case in which no parasitic forms were detected.

**Figure 2:**
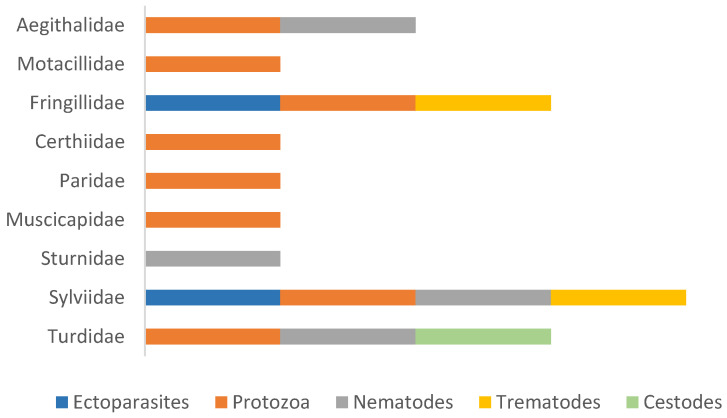
Distribution of the detected parasitic groups by passerine family.

Upon completing the analysis of all samples, the identified parasites were categorized into three main groups: ectoparasites, which were detected in two of the passerines (2/17; 12%); protozoan endoparasites, found in twelve of the passerines (12/17; 71%); and helminth endoparasites including nematodes, trematodes and cestodes, present in ten of the passerines (10/17; 59%),

The presence and distribution of these parasites varied across different bird families, as illustrated in Figure 2. The Sylviidae family exhibited the highest diversity of parasitic groups. Cestodes were found only in the Turdidae family, while protozoa were absent in the Sturnidae family. Additionally, the Fringillidae and Sylviidae families were the only ones to host both ectoparasites and trematodes within this sample.

**Figure 3:**
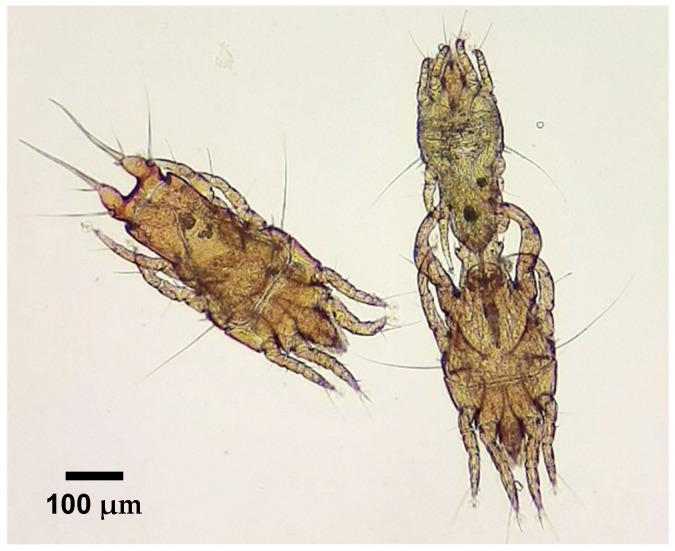
Female (**on the left**), male (**on the right**, below) and tritonymph (**on the right**, above) of *Monojoubertia microhylla* on a common chaffinch (*Fringilla coelebs*).

**Figure 4:**
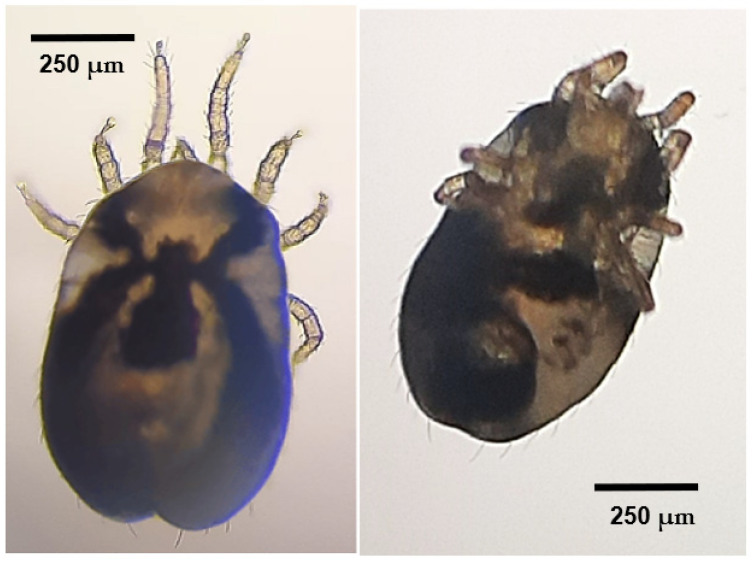
Dorsal view (**on the left**) and ventral view (**on the right**) of *Ornithonyssus* sp. on a Eurasian blackcap (*Sylvia atricapilla*).

At the start of the necropsies of two of the passerines in the sample, during the external examination, ectoparasites were detected. In one case, adult specimens of both sexes and tritonymphs of *Monojoubertia microhylla* (Figure 3) were observed, and other parasites were identified as belonging to the genus *Ornithonyssus* (Figure 4).

**Figure 5:**
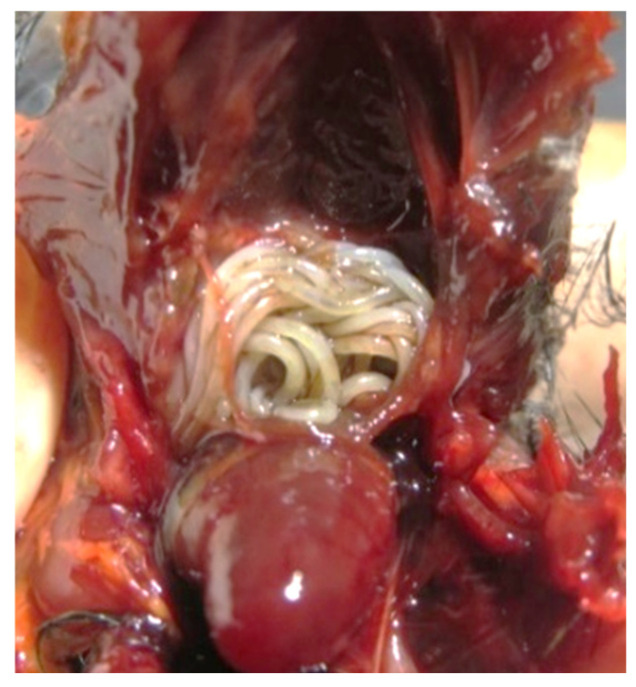
Parasites of the genus *Diplotriaena* sp. in the air sacs of a Eurasian blackcap (*Sylvia atricapilla*).

**Figure 6:**
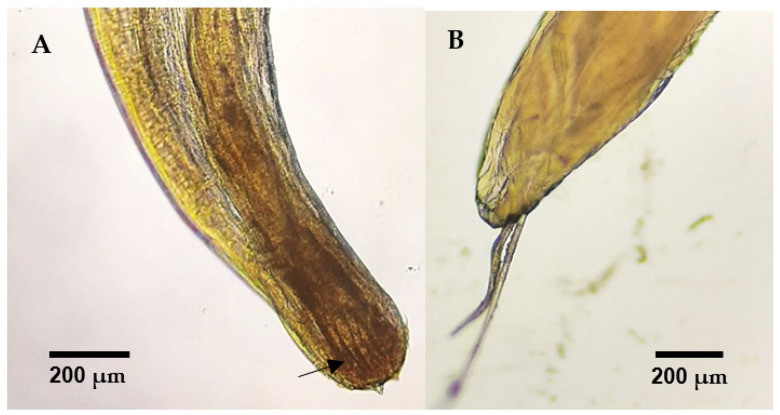
(**A**) Anterior end of *Diplotriaena* sp., where the trident can be observed (arrow). (**B**) Spicules at the posterior end of a *Diplotriaena* sp. male.

**Figure 7:**
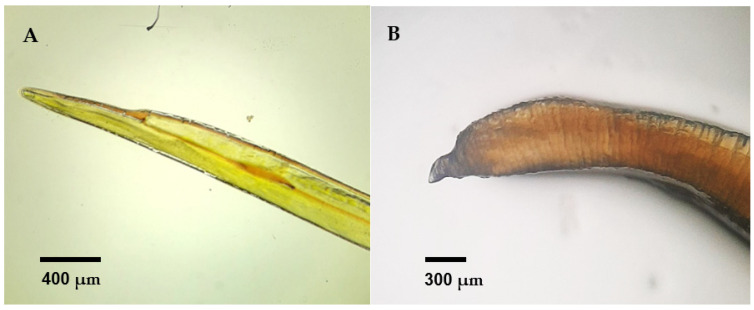
(**A**) Anterior and posterior ends of *Serratospiculum* sp. from a song thrush (*Turdus philomelos*). (**B**) Posterior end of Porrocaecum sp. from a Eurasian blackbird (*Turdus merula*).

Among the total number of necropsies performed, 35% (6/17) were considered positive for the presence of macroscopic adult parasitic forms, specifically nematodes (Figure 5). Parasites identified as *Diplotriaena* spp. (Figure 6) and *Serratospiculum* sp. (Figure 7) were detected in the air sacs, while a specimen from the genus *Porrocaecum* (Figure 7) was found in the intestine.

**Figure 8:**
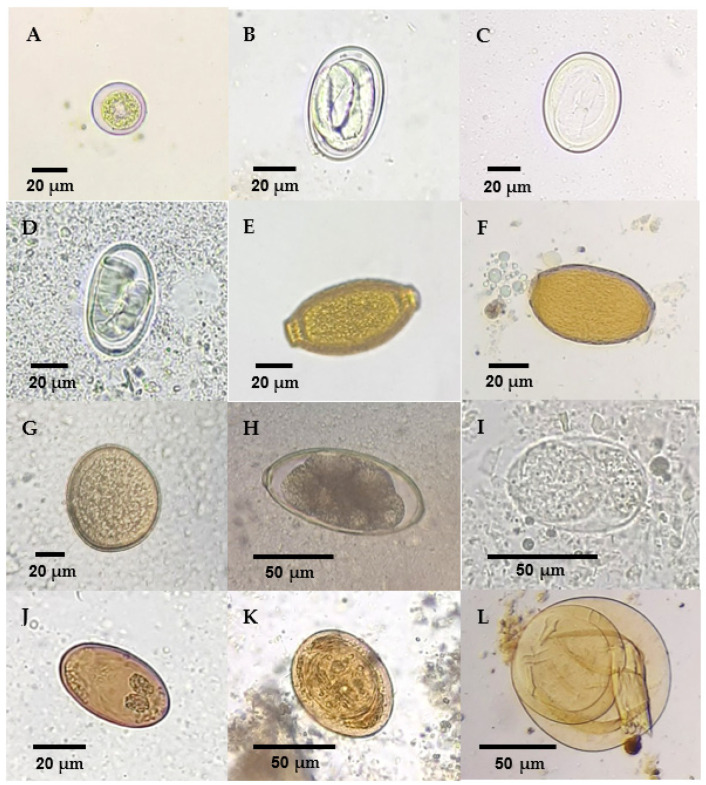
Immature parasitic forms detected by coprological techniques: (**A**) Unsporulated oocyst of coccidia. (**B**–**D**) Eggs of *Diplotriaena* spp. (**E**,**F**) Eggs of *Capillaria* spp. (**G**) Egg of *Porrocaecum* sp. (**H**) Egg of *Syngamus* sp. (**I**) Egg of *Strongyloides* sp. (**J**) Egg of *Brachylecithum* sp. (**K**,**L**) Eggs of cestodes.

Through direct examination/fecal smear, eleven samples were found to be positive for coccidia (65%), three for *Diplotriaena* spp. (18%), one for *Capillaria* spp. (6%), one for *Porrocaecum* sp. (6%), one for *Syngamus* sp. (6%), one for *Strongyloides* sp. (6%), one for *Brachylecithum* sp. (6%) and two for cestodes (12%) (Figure 8).

Using the Willis flotation technique, twelve samples were found to be positive for coccidia (71%), one for *Diplotriaena* spp. (6%) and one for *Capillaria* spp. (6%) (Figure 8).

When the sedimentation technique (by centrifugation) was applied, eight samples tested positive for coccidia (47%), three for *Diplotriaena* spp. (18%), one for *Capillaria* spp. (6%), one for *Porrocaecum* sp. (6%) and one for *Syngamus* sp. (6%). Additionally, two samples were confirmed positive for *Brachylecithum* sp. (12%) and two for cestodes (12%) (Figure 8).

**Figure 9:**
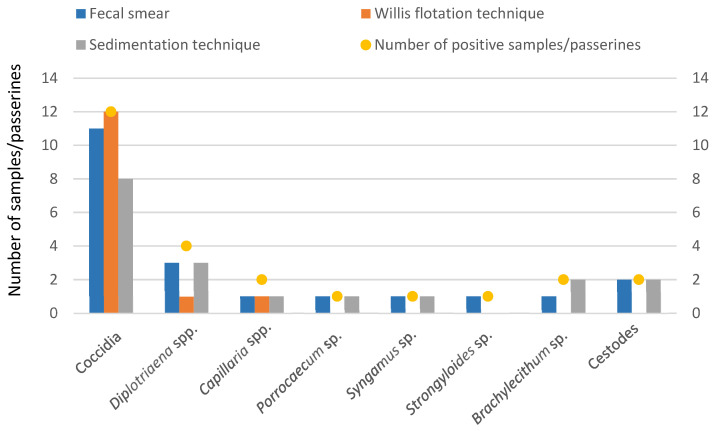
Comparison of qualitative coprological techniques in the detection of positive parasitological samples.

As shown in Figure 9, we compared the results of each qualitative coprological test in detecting the parasites identified in the study.

The positive samples for coccidia were detected by the Willis flotation technique, with 92% (11/12) identified through direct examination and only 67% (8/12) detected by the sedimentation technique.

Regarding the eggs of *Diplotriaena* spp., they were detected in 75% (3/4) of cases by direct examination and the sedimentation technique, whereas the Willis flotation technique only revealed 25% (1/4) of the positive samples for this parasite. *Capillaria* spp. was identified in half (1/2) of the passerines positive for this parasite, regardless of the qualitative coprological test used. Direct examination and the sedimentation technique revealed all positive samples for the nematodes *Porrocaecum* sp. and *Syngamus* sp. (1/1), while the Willis flotation technique failed to enable the detection of their eggs. Finally, the eggs of *Strongyloides* sp. in the sample were evidenced by direct examination.

The eggs of *Brachylecithum* sp. were found in all passerines positive for trematodes using the sedimentation technique but only in 50% (1/2) of these using direct examination and none using the Willis flotation test. Cestode eggs were detected in both (2/2) samples that were positive for these parasites by direct examination and the sedimentation technique, but no cases were evidenced by the Willis flotation technique.

When comparing the adult forms of helminths detected during necropsies with the eggs observed using coprological techniques, it was noted that in 27% (4/15) of cases, the eggs were consistent with the adult forms present. However, adult parasites of *Serratospiculum* sp. and *Porrocaecum* sp. were identified in 13% (2/15) of cases, without any eggs from these genera appearing in the coprological examinations. Additionally, in 60% (9/15) of the cases, eggs were detected, but their adult forms were not confirmed during necropsy.

Table 1 highlights the parasites detected by each technique, matching them to the corresponding passerine from the sample.

### 3.3. Quantitative Parasitological Results

Of the 17 intestinal content samples analyzed using the McMaster method, 71% (12/17) showed countable parasitic forms. However, in 20% (3/15) of the passerines that tested positive using qualitative coprological techniques, no eggs or oocysts were detected in the McMaster chamber.

Given that the detection limit for this quantitative method is 50 eggs per gram (EPG) or oocysts per gram (OPG), it was determined that 47% (7/15) of the samples positive for nematodes in qualitative coprology were below this limit for one or more parasite genera; thus, their values were considered to be under 50 (Table 2).

Coccidia were countable in 100% (12/12) of the passerines that tested positive for them using qualitative techniques (Table 2).

**Figure 10:**
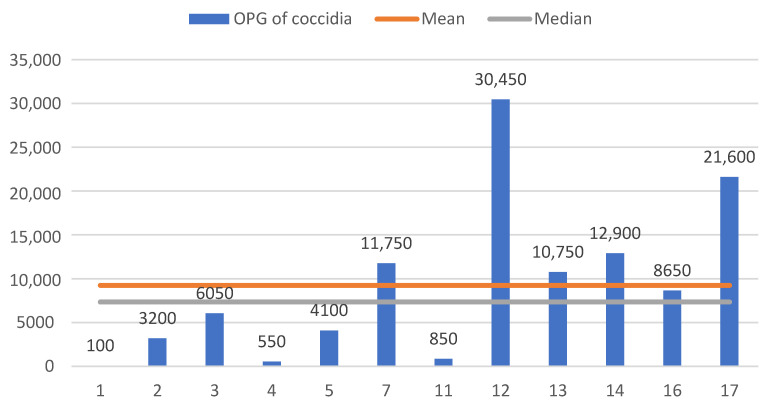
Results of coccidial oocyst counting using the McMaster method.

Among the 12 passerines positive for coccidia (Figure 10), the observed oocyst counts per gram of feces ranged from 100 to 30,450 OPG, with the Turdidae family and the coal tit recording the highest counts. The sample had an average of 9245.8 OPG, but since the data did not follow a normal distribution and there was an outlier value (30,450 OPG), the median of 7350 OPG was considered (Figure 10).

## 4. Discussion

This study was conducted with a sample of wild passerines admitted to GREFA. Given the sample size of 17 individuals, the study included a broad diversity of Passeriformes species, specifically 12, which was entirely dependent on the admissions to the center during October, November and December. It is relevant to note that the number of birds admitted to rehabilitation centers is generally lower during autumn and winter, increasing in spring and peaking during summer, as indicated by Mullineaux and Pawson (2023) [32].

We acknowledge that the sampling in this study was limited to the passerines admitted to GREFA, which may introduce a degree of bias due to specific factors influencing admission rates. Certain species, such as those in the Turdidae family, are more frequently admitted to centers because of their ecological characteristics such as size, foraging behavior and proximity to human settlements [32,33]. However, the diversity of species included in the study occurred naturally, as all 12 species are characteristic of the local avifauna and are expected to occur in the region during autumn and winter, either as residents or as migrants. This overlap between the species admitted and the local passerine diversity suggests that, while the sampling reflects the limitations inherent to rehabilitation center data, it still provides a relevant representation of the passerines in the area during the study period.

In terms of parasitological data, it was observed that the vast majority, 16 out of 17 (94.1%) of the sampled passerines, tested positive for the presence of parasites. Although there are studies [12,23] addressing parasitism in these wild birds, they focus on specific parasitic groups, and there are no comparative values for a broader analysis.

In this study, ectoparasites were detected in only two of the passerines in the sample (2/17; 12%), a lower percentage than reported in other studies. A study conducted in Turkey between 2015 and 2019 analyzed 188 injured wild birds and found that 88 (46.8%) were infected with external parasites [34]. Another study identified at least one ectoparasite species in 114 of 266 (43%) wild passerines analyzed in the Azores Archipelago [35]. As such, the lower detection rate of ectoparasites in our study is likely due to the lack of specific methods for collecting them, such as modified fumigation chambers, the use of ethanol-impregnated cotton on feathers, or simply the use of a white working surface [23,36].

In contrast, protozoan endoparasites were found in 12 of the passerines (12/17; 71%), and helminth endoparasites were present in 10 of the passerines (10/17; 59%), showing higher infection rates than external parasites.

Two studies reported different percentages of birds infected with coccidia, the only protozoa detected in this study. Assam et al. (2020) [37], who examined 357 birds, including some in captivity, found that 39.1% of wild birds were infected with coccidia. On the other hand, Dolnik et al. (2010) [38] found a higher percentage of coccidia in passerines, at 66.6%, which is closer to the 71% observed in this study.

Indeed, while the prevalence of coccidia in wild passerine species has been documented, such observations are often incidental or based on small sample sizes [39] or do not account for variations in oocyst excretion throughout the day by passerines, leading to varying results that may not accurately reflect population-wide situations [40]. Additionally, Keckeisen et al. (2024) [41] found *Isospora* sp. not only in the intestine but also in other organs, emphasizing the presence of extraintestinal stages in passerines and underscoring the need for additional diagnostic methods, such as blood smears (in live birds) and histological sections of the spleen, liver, heart and lungs for accurate detection.

Although helminth endoparasites have only recently become a focus of studies in passerine hosts within rehabilitation centers, there remains a significant lack of available data. A study conducted in southern Portugal collected adult parasites from the gastrointestinal tract of 22 wild bird specimens that died at the Ria Formosa Wildlife Rehabilitation and Research Center [42]. The overall infection level was 54.5% (12/22), with nematodes being the most frequent helminths, as observed in this study [42].

Additionally, Parsa et al. (2023) [43] conducted a survey of 755 wild passerines from England, Wales and Ireland to identify intestinal helminths and protozoa and study their epidemiology. As we did in the present study, they observed coccidia, nematodes, trematodes and cestodes, but not acanthocephalans, though these have been identified in passerines before. The presence of parasites also varied among bird families and it is important to note both similarities and disparities between the results described and those of this study, as factors such as season, presence of intermediate hosts, sex and age of birds can influence parasitic prevalence, abundance and richness [44]. The families Sylviidae and Turdidae were also infected with both helminths and coccidia, whereas the family Sturnidae had only helminths. However, trematodes were identified in the Fringillidae family and both parasitic groups in the Aegithalidae family.

Indeed, dietary habits and feeding behaviors influence the parasitic fauna of birds [7]. Passerines, especially most of the thrushes (Turdidae), are more exposed to trophically transmitted eggs and oocysts due to their diverse diet and foraging habits, which may explain a higher variety and incidence of parasites. Additionally, omnivorous passerines, such as starlings (Sturnidae), tend to be more heavily parasitized by helminths than granivorous species, such as sparrows (Passeridae) and finches (Fringillidae) [43]. Variations in parasitism among passerine families may also be attributed to coevolutionary adaptations caused by geographical and climatic differences, as well as the host’s immune status and genetic resistance [43].

The coprological techniques used in this study are well established in the literature, with widespread application, reliability and practicality in detecting parasites in various animal species, including birds [8]. They can be easily applied in wildlife rehabilitation centers, aligning with the practical constraints of such facilities.

Direct examination was selected for its simplicity, effectiveness with small sample sizes [10] and suitability for quick assessment. Additionally, the experience of the veterinarians at the center recommended this method as reliable for wild birds. Although it provides an overview of the type and intensity of parasitism in the host, its limitations must be acknowledged, as it is considered a less sensitive technique, more suitable for detecting higher parasite intensities [8].

Flotation and sedimentation techniques were utilized to concentrate parasitic forms, thereby improving detection sensitivity and accuracy. A saturated magnesium sulfate solution was chosen due to its ideal density (between 1.2 and 1.3 g/mL), facilitating effective flotation without being too aggressive on the parasitic forms [8].

Finally, the McMaster method is a widely recognized quantitative coprological technique in veterinary medicine, serving as both a diagnostic tool and a means to monitor antiparasitic therapies or to guide group prophylactic decisions [8,12]. This method was included for its reliability and proven validation in parasitological studies, particularly in quantifying *Isospora* oocysts in passerines [12], making it an essential addition to the diagnostic protocol.

The comparative analysis of the qualitative parasitic detection methods in this study highlighted the effectiveness of the Willis flotation test in identifying coccidia, as it was the only method used in the limited coprological studies of passerines. However, the importance of performing direct examination or sedimentation techniques as complementary methods became evident, as these tests revealed other genera of parasites in the samples that had not been detected by the flotation test.

For instance, Stanicka et al. (2021) [22] mention that the saturated magnesium sulfate flotation method may fail to detect low-intensity infections of *Diplotriaena* sp., and Becker et al. (2016) [10] infer from their research that fecal samples are more reliably identified using a combined sedimentation and flotation method.

To date, there are no records of studies that have performed coprological analyses of passerine intestinal content. Therefore, it is crucial to explore potential adaptations of these methods, especially in selecting the most suitable saturated solution, as its density and intrinsic characteristics may significantly impact the results [45].

The most frequent parasites in this study were *Diplotriaena* spp. and coccidia. Various other studies, such as that of Parsa et al. (2023) [43], also report coccidia and nematodes (e.g., *Syngamus* sp. and *Capillaria* sp.) as the most prevalent. However, the main discrepancy lies in the lack of identification and subsequent exclusion of the genus *Diplotriaena* from the list of most common parasites, a trend often observed, particularly in works not focusing specifically on this parasite.

This absence may be attributed to the scarcity of reports on respiratory parasites in passerines, resulting in a general lack of awareness. Additionally, the neglect of research on the genus *Diplotriaena* and its hosts [22], combined with the need to observe adult forms for accurate genus identification, may contribute to this disparity. This underscores the importance of performing necropsies to ensure a comprehensive understanding of parasitic infections and to accurately identify all parasitic genera affecting Passeriformes.

The techniques employed revealed common parasites such as *Capillaria* spp. and *Syngamus* sp., as well as genera newly recorded in wild passerines on the Iberian Peninsula, including *Serratospiculum* sp. and *Ornithonyssus* sp., and genera reported for the second time, such as *Porrocaecum* sp. and *Brachylecithum* sp. Additionally, *Diplotriaena* spp. was identified, having not been documented in similar contexts since 1979 [45]. These findings highlight the still poorly understood distribution of these genera across Europe and underscore the need for further research.

It is important to note that the majority of the parasites identified in this study rely on intermediate hosts that are present in the diet of the sampled passerines [33]. However, we also identified parasites with direct life cycles, which may have a more significant impact in certain situations, particularly during the rehabilitation process in wildlife centers or during the winter months.

The presence, prevalence and overall parasitism should, therefore, always be interpreted with consideration of the context in which the bird is found. Specifically, geographic location and seasonality factors, such as temperature, humidity, food availability, migration and reproductive activity [46], play a significant role. In this study, data were collected during the autumn and winter months, periods in which parasitic dynamics can differ from spring and summer. Seasonal variations in parasitism can influence the results, underscoring the importance of careful interpretation when comparing studies conducted in different seasons.

In the present study, eggs whose adult forms could not be confirmed during the necropsy were identified in 60% (9/15) of cases, highlighting the need to improve sample processing. The main challenge lies in handling the tissues and structures of passerines during necropsy, particularly when opening the intestine to collect its contents. This difficulty arises not only from the fragility of the tissues but also from the lack of access to microsurgical instruments for use in conducting necropsies, which are unavailable in some rehabilitation centers, including GREFA.

In this study, egg and oocyst counts were conducted using the quantitative McMaster method. In fact, no nematode eggs were detected, which indicates that in passeriform birds testing positive in qualitative techniques for a given parasite, the counts were below the detection limit of the method and therefore under 50 EPG. It is important to note that counts below 50 EPG are not equivalent to zero; for instance, a sample with 30 EPG still represents an important result. This highlights the potential utility of alternative methods, such as the Mini-FLOTAC, which may provide greater sensitivity and precision in detecting low-intensity parasite infections [47].

Coccidia oocysts were the most abundant among the parasites identified in this study, similar to the findings of Parsa et al. (2023) [43], who used the McMaster method on fecal samples from 755 wild passerines and found that *Isospora* sp. had the highest overall abundance, while trematodes had the lowest. We are focusing on *Isospora* for comparison because it is the most prevalent and significant coccidian genus in Passeriformes, surpassing *Eimeria* [8], and due to the scarcity of recent studies offering seasonal data on coccidia prevalence and abundance in these birds.

In the present work, oocyst counts per gram of intestinal contents ranged from 100 to 30,450 OPG, with a sample mean of 9245.8 OPG and a median of 7350 OPG. Compared to the study by Parsa et al. (2023) [43], which reported an average oocyst abundance of *Isospora* sp. of 6080 ± 2197.4 OPG in winter, our research obtained similar but higher values.

Several factors can influence coccidial oocyst counts in birds, with season, habitat diversity and age being strongly influential [39,43]. It was observed that passerines sampled in winter had a higher abundance of *Isospora* sp., closely aligning with the results of this study, whereas spring values were considerably lower, affecting the overall abundance of these parasitic forms (1485.2 ± 500.51 OPG) [43]. These variations are significant and should be considered in data interpretation.

The higher oocyst counts in this study were likely due to the sample consisting of debilitated wild passerines that were handled and kept in captivity, which may have disrupted the host–parasite balance and promoted coccidial replication. Although counts of up to 38,940 oocysts per gram (OPG) in a single bird have been reported [47], the 30,450 OPG count appeared as an outlier in our study’s general analysis. However, it was included in the dataset because it is considered a valid value that contributes to understanding the variability within the sample. This highlights the need for further research to better understand these variations and address sample size limitations.

The higher abundance of coccidial oocysts in Turdidae, as observed in both this study and Parsa et al. (2023) [43], reinforces the hypothesis that foraging habits may play a significant role in parasitic infection levels [39].

In wildlife recovery centers, coccidia are particularly important parasites, as transportation, handling, confinement, injury and illness facilitate the transmission of large quantities of oocysts, due to an imbalance in the host immune system [30]. Additionally, coprological analyses are not routinely performed without clinical suspicion, leading to potential failures in coccidial identification and quantification and subsequent reintroduction of animals into naive environments [30]. Therefore, it is crucial to minimize human interactions and confinement, ensure proper cleaning of facilities (for example, with quaternary ammonium compounds), optimize ventilation [48] and, importantly, raise awareness of this issue.

This pilot study aimed not only to detect parasites in wild passerines but also to evaluate the outcomes of necropsies, coprological techniques and sample collection methods, particularly for intestinal contents, as no previous studies have addressed these aspects. We focused on methods that are practical for rehabilitation centers—quick and cost-effective—highlighting the importance of these features in encouraging the use of the methods. We propose implementing the improvements detailed in the article, repeating the study with a larger sample and incorporating additional diagnostic tests also mentioned. Our goal is to gain a deeper understanding of passerines and the parasites affecting them to improve their management and treatment, with the eventual aim of enhancing in vivo parasitological testing as well.

## 5. Conclusions

This study emphasizes the critical role of thorough necropsies and diagnostic parasitological methods in examining wild passerines. It highlights the first use of intestinal content coprology in these species, a cost-effective and timely method that enhances post mortem diagnostics and promotes future research. The combination of sedimentation and flotation techniques showed promise, though improvements in sample collection and processing are needed.

This study also provides a comprehensive analysis of the parasitofauna of wild passerines, revealing a high parasitic prevalence of 94.1% (16/17). Key findings include the frequent detection of *Diplotriaena* spp. and coccidia, as well as the first report of *Serratospiculum* sp. on the Iberian Peninsula. Wildlife rehabilitation centers have proven to be valuable sources of information on these birds, providing crucial insights into their parasitic infections and health, making it essential to invest in practical diagnostic methods to enhance their capacity for effective wildlife management and monitoring.

## Figures and Tables

**Table 1:** Results of qualitative parasitic detection methods.

Sample Number	Species of Passeriforms	Familly of Passeriforms	Ectoparasites	Adult Forms (Necropsy)	Fecal Smear	Willis Flotation Technique	Sedimentation Technique
1	*Aegithalos caudatus*	Aegithalidae	-	*Diplotriaena* spp.	-	Coccidia	*Diplotriaena* spp.
2	*Anthus pratensis*	Motacillidae	-	-	Coccidia	Coccidia	Coccidia
3	*Carduelis chloris*	Fringillidae	-	-	Coccidia	Coccidia	*Brachylecithum* sp., Coccidia
4	*Certhia brachydactyla*	Certhiidae	-	-	Coccidia	Coccidia	-
5	*Erithacus rubecula*	Muscicapidae	-	-	Coccidia	Coccidia	-
6	*Fringilla coelebs*	Fringillidae	*Monojoubertia microhylla*	-	-	-	-
7	*Parus ater*	Paridae	-	-	Coccidia	Coccidia	Coccidia
8	*Passer domesticus*	Passeridae	-	-	-	-	-
9	*Sturnus unicolor*	Sturnidae	-	*Diplotriaena* spp.	*Diplotriaena* spp.	-	*Diplotriaena* spp.
10	*Sylvia atricapilla*	Sylviidae	*Ornithonyssus* sp.	*Diplotriaena* spp.	*Diplotriaena* spp.; *Brachylecithum* sp.	*Diplotriaena* spp.	*Diplotriaena* spp.; *Brachylecithum* sp.
11	*Sylvia atricapilla*	Sylviidae	-	*Diplotriaena* spp.	*Diplotriaena* spp.; Coccidia	Coccidia	-
12	*Turdus merula*	Turdidae	-	*Porrocaecum* sp.	Coccidia	Coccidia	Coccidia
13	*Turdus merula*	Turdidae	-	-	Cestode; Coccidia	Coccidia	Cestode, Coccidia
14	*Turdus merula*	Turdidae	-	-	Coccidia	Coccidia	Coccidia
15	*Turdus philomelos*	Turdidae	-	*Serratospiculum* sp.	*Capillaria* spp.; Cestode	-	*Capillaria* spp., Cestode
16	*Turdus philomelos*	Turdidae	-	-	*Porrocaecum* sp.; *Syngamus* sp.; Coccidia	Coccidia	*Porrocaecum* sp.; *Syngamus* sp.; Coccidia
17	*Turdus philomelos*	Turdidae	-	-	*Strongyloides* sp., Coccidia	*Capillaria* spp.; Coccidia	Coccidia

**Table 2:** Results of parasitic form counts by the McMaster method.

		EPG and OPG Counts by the McMaster Method					
Sample Number	Species of Passeriforms	Coccidia	*Diplotriaena* spp.	*Capillaria* spp.	*Porrocaecum* sp.	*Syngamus* sp.	*Strongyloides* sp.
1	*Aegithalos caudatus*	100	<50	0	0	0	0
2	*Anthus pratensis*	3200	0	0	0	0	0
3	*Carduelis chloris*	6050	0	0	0	0	0
4	*Certhia brachydactyla*	550	0	0	0	0	0
5	*Erithacus rubecula*	4100	0	0	0	0	0
6	*Fringilla coelebs*	0	0	0	0	0	0
7	*Parus ater*	11,750	0	0	0	0	0
8	*Passer domesticus*	0	0	0	0	0	0
9	*Sturnus unicolor*	0	<50	0	0	0	0
10	*Sylvia atricapilla*	0	<50	0	0	0	0
11	*Sylvia atricapilla*	850	<50	0	0	0	0
12	*Turdus merula*	30,450	0	0	0	0	0
13	*Turdus merula*	10,750	0	0	0	0	0
14	*Turdus merula*	12,900	0	0	0	0	0
15	*Turdus philomelos*	0	0	<50	0	0	0
16	*Turdus philomelos*	8650	0	0	<50	<50	0
17	*Turdus philomelos*	21,600	0	<50	0	0	<50

## Data Availability

The data presented in this study are available on request from the corresponding authors. The data are not publicly available.

## References

[1] Schmitt C.J., Edwards S.V. (2022). Passerine birds. Curr. Biol. Mag..

[2] Atkinson C.T., Thomas N.J., Hunter D.B. (2008). Parasitic Diseases of Wild Birds.

[3] Carrera-Játiva P.D., Morgan E.R., Barrows M., Jiménez-Uzcátegui G., Armijos Tituaña J.R. (2020). Free-ranging avifauna as a source of generalist parasites for captive birds in zoological settings: An overview of parasite records and potential for cross-transmission. J. Adv. Vet. Anim. Res..

[4] Sajid M., Ehsan N. (2017). Insect ectoparasites on wild migratory birds: A review. Anim. Sci. J..

[5] Krone O., Cooper J.E. (2002). Parasitic diseases in Birds of prey-Health & Disease.

[6] Souchay G., Gauthier G., Pradel R. (2013). Temporal variation of juvenile survival in a long-lived species: The role of parasites and body condition. Oecologia.

[7] Leung T.L., Koprivnikar J. (2016). Nematode parasite diversity in birds: The role of host ecology, life history and migration. J. Anim. Ecol..

[8] Conboy G., Zajac A. (2012). Veterinary Clinical Parasitology.

[9] Madeira de Carvalho L. (2002). Epidemiologia e controlo da estrongilidose em diferentes sistemas de produção equina em Portugal. Doctoral Thesis.

[10] Cunha I.P., Rodrigues Junior O.M. (2021). Avaliação da sensibilidade dos métodos direto à fresco e Hoffman para *Ascaris lumbricoides*. Res. Soc. Dev..

[11] Becker A.C., Kraemer A., Epe C., Strube C. (2016). Sensitivity and efficiency of selected coproscopical methods—Sedimentation, combined zinc sulfate sedimentation-flotation and McMaster method. Parasitol. Res..

[12] Vadlejch J., Petrtýl M., Lukešová D., Čadková Z., Kudrnáčová M., Jankovská I., Langrová I. (2012). The concentration McMaster technique is suitable for quantification of coccidia oocysts in bird droppings. Pakistan Vet. J..

[13] Weisbroth S.H. (1960). The Differentiation of *Dermanyssus gallinae* from *Ornithonyssus sylviarum*. Avian. Dis..

[14] Levin N.L. (1961). Life History Studies on *Porrocaecum ensicaudatum* (Nematoda), an Avian Nematode. Ph.D. Thesis.

[15] Proctor H., Owens I. (2000). Mites and Birds: Diversity, parasitism and coevolution. Trends Ecol. Evol..

[16] Mobedi I., Sehhatisabet M.E., Razmjou E., Shafiei S. (2006). First record of Diplotriaena henryi Blanc, 1919 from the coal tit, *Parus ater* with new report from the great tit, *Parus major* in the Middle East. Helminthologia.

[17] Palma A.D., Giangaspero A., Cafiero M.A., Germinara G.S. (2012). A gallery of the key characters to ease identification of *Dermanyssus gallinae* (Acari: Gamasida: Dermanyssidae) and allow differentiation from *Ornithonyssus sylviarum* (Acari: Gamasida: Macronyssidae). Parasit Vectors.

[18] Königová A., Molnár L., Hrčková G., Várady M. (2013). The first report of serratospiculiasis in great tit (*Parus major*) in Slovakia. Helminthologia.

[19] Morais J., Souza D., Gallas M., Silveira E., Périco E. (2018). *Diplotriaena delirae* Pinto & Noronha,1970 (Nematoda, Diplotriaenidae) in *Pitangus sulphuratus* (Linnaeus, 1766) (Passeriformes, Tyrannidae) from southern Brazil. Check List..

[20] Hong E.J., Ryu S.Y., Chae J.S., Kim H.C., Park J., Cho J.G., Choi K.S., Yu D.H., Park B.K. (2019). Description of *Diplotriaena manipoli* (nematoda: Diplotriaenoidea) detected in the body cavity of *Garrulus glandarius brandtii* from Republic of Korea. J. Vet. Clin..

[21] Li L., Scholz T. (2019). Redescription of *Porrocaecum semiteres* (Zeder, 1800) (Nematoda: Ascaridida) from the Song Thrush *Turdus philomelos* (Passeriformes: Turdidae). Acta Parasitol..

[22] Stanicka A., Zając K.S., Jefimow M., Wojciechowski M.S. (2021). *Diplotriaena obtusa* (Nematoda: *Filariidae*) infection in first-year *Sylvia atricapilla* from Poland—Molecular evidence. Eur. Zool. J..

[23] Tomás A.F. (2021). Passeriformes Colonization and Related Ectoparasites in Insular and Mainland Populations. Ph.D. Thesis.

[24] Carney W.P. (1970). *Brachylecithum mosquensis*: Infections in vertebrate, molluscan and arthropod hosts. Trans. Am. Microsc. Soc..

[25] Gomez P.I., López R.R. (1979). Parasitación por helmintos de las aves de la provincia de Granada. Ars. Pharm..

[26] Wharton D.A. (1979). The structure of the egg-shell of *Porrocaecum ensicaudatum* (Nematoda: Ascaridida). Int. J. Parasitol..

[27] Taylor M., Coop R., Wall R. (2016). Parasitologia Veterinária.

[28] Veiga I.B., Schediwy M., Hentrich B., Frey C.F., Marreros N., Stokar-Regenscheit N. (2017). Serratospiculosis in captive peregrine falcons (*Falco peregrinus*) in Switzerland. J. Avian Med. Surg..

[29] Wettere A.J., Kurz J.P., Wilhelm A., Ipsen J.D. (2018). Opisthotonos and unilateral internal hydrocephalus associated with aberrant migration of *Serratospiculum* sp. or *Serratospiculoides* sp. in a prairie falcon. J. Vet. Diagn. Investig..

[30] Cardozo S.V., Berto B.P., Caetano I., Thomás A., Santos M., Fonseca I.P., Lopes C.W. (2019). Coccidian parasites from birds at rehabilitation centers in Portugal, with notes on *Avispora bubonis* in Old World. Rev. Bras. Parasitol. Vet..

[31] Oliveira H.G., Santos R.C.d., Lopes C.T., Souza A.I., Almeida D.V., Scalercio S.R., Viott A.M., Domingues S.F., Salvarani F.M. (2023). Airsacculitis caused by enterobacteria and occurrence of eggs of the superfamily Diplotriaenoidea in feces of tropical screech owl (*Megascops choliba*) in the Amazon biome. Animals.

[32] Mullineaux E., Pawson C. (2023). Trends in Admissions and Outcomes at a British Wildlife Rehabilitation Centre over a Ten-Year Period (2012–2022). Animals.

[33] Svensson L., Mullarney K., Zetterström D. (2022). Guia de Aves.

[34] Girişgin O., Girişgin A.O., Cimenlikaya N., Saygin B. (2023). A survey of the ectoparasites found on wild birds in northwest Turkey. Indian J. Anim. Res..

[35] Oslejskova L., Kounkova S., Gustafsson D.R., Resendes R., Rodrigues P., Literak I., Sychra O. (2020). Insect ectoparasites from wild passerine birds in the Azores Islands. Parasite.

[36] Vila-Viçosa M.J., Reis B., Godinho C., Catarino L., Cortes H. Occurrence and diversity of feather mites on Passeriformes on the South of Portugal. Proceedings of the IX Congresso de Ornitologia da SPEA and VI Congresso Ibérico de Ornitologia.

[37] Assam A., Salamatu A., Abdu P., Ezealor A. (2020). Endo-parasites of apparently healthy wild birds in Kaduna State, Nigeria. Annu. Res. Rev. Biol..

[38] Dolnik O.V., Dolnik V.R., Bairlein F. (2010). The effect of host foraging ecology on the prevalence and intensity of coccidian infection in wild passerine birds. Ardea.

[39] Knight A., Ewen J.G., Brekke P., Santure A.W. (2018). The evolutionary biology, ecology and epidemiology of coccidia of passerine birds. Adv. Parasitol..

[40] Biard C., Monceau K., Teixeira M., Motreuil S., Bettencourt-Amarante S., Develay L., Moreau J. (2022). Coccidial oocyst release: Once a day or all day long? Tropical bird hosts shed new light on the adaptive significance of diurnal periodicity in parasite output. Parasitology.

[41] Keckeisen C., Šujanová A., Himmel T., Matt J., Nedorost N., Chagas C.R., Weissenböck H., Harl J. (2024). *Isospora* and *Lankesterella* Parasites (Eimeriidae, Apicomplexa) of Passeriforme Birds in Europe: Infection Rates, Phylogeny and Pathogenicity. Pathogens.

[42] Tomás A., Rebelo M.T., Fonseca I.P. (2017). Occurrence of helminth parasites in the gastrointestinal tract of wild birds from Wildlife Rehabilitation and Investigation Centre of Ria Formosa in southern Portugal. Vet. Parasitol. Reg. Stud. Rep..

[43] Parsa F.R., Bayley S., Bell F., Dodd S., Morris R., Roberts J., Wawman D., Clegg S.R., Dunn J.C. (2023). Epidemiology of protozoan and helminthic parasites in wild passerine birds of Britain and Ireland. Parasitology.

[44] Cardells-Peris J., Gonzálvez M., Ortega-Porcel J., Ybáñez M.R., Martínez-Herrero M.C., Garijo-Toledo M.M. (2020). Parasitofauna survey of song thrushes (*Turdus philomelos*) from the eastern part of Spain. Parasitol. Int..

[45] Cringoli G., Rinaldi L., Veneziano V., Capelli G., Scala A. (2004). The influence of flotation solution, sample dilution and the choice of McMaster slide area (volume) on the reliability of the McMaster technique in estimating the fecal egg counts of gastrointestinal strongyles and *Dicrocoelium dendriticum* in sheep. Vet. Parasitol..

[46] Massey J.G. (2003). Diseases and medical management of wild passeriformes. Semin. Avian. Exotic. Pet. Med..

[47] Lozano J., Anaya A., Rinaldi L., Cringoli G., Gomes L., Oliveira M., Paz-Silva A., Rebelo M.T., Carvalho L.M. (2021). Diagnosis of coccidiosis by *Eimeria* spp. in free-range chickens using Mini-FLOTAC and McMaster techniques-preliminary results. Sci. Parasitol..

[48] Attree E., Sanchez-Arsuaga G., Jones M., Xia D., Marugan-Hernandez V., Blake D., Tomley F. (2021). Controlling the causative agents of coccidiosis in domestic chickens; an eye on the past and considerations for the future. CABI Agric. Biosci..

